# Feature elimination and stacking framework for accurate heart disease detection in IoT healthcare systems using clinical data

**DOI:** 10.3389/fmed.2024.1362397

**Published:** 2024-05-22

**Authors:** Wang Jian, Jian Ping Li, Amin Ul Haq, Shakir Khan, Reemiah Muneer Alotaibi, Saad Abdullah Alajlan, Md Belal Bin Heyat

**Affiliations:** ^1^School of Artificial Intelligence, Neijiang Normal University, Neijiang, Sichuan, China; ^2^School of Computer Science and Engineering, University of Electronic Science and Technology of China, Sichuan, Chengdu, China; ^3^University Centre for Research and Development, Department of Computer Science and Engineering, Chandigarh University, Mohali, India; ^4^College of Computer and Information Sciences, Imam Mohammad Ibn Saud Islamic University (IMSIU), Riyadh, Saudi Arabia; ^5^CenBRAIN Neurotech Center of Excellence, School of Engineering, Westlake University, Hangzhou, Zhejiang, China

**Keywords:** heart disease, machine learning, classification, stacking, healthcare

## Abstract

**Introduction:**

Heart disease remains a complex and critical health issue, necessitating accurate and timely detection methods.

**Methods:**

In this research, we present an advanced machine learning system designed for efficient and precise diagnosis of cardiac disease. Our approach integrates the power of Random Forest and Ada Boost classifiers, along with incorporating data pre-processing techniques such as standard scaling and Recursive Feature Elimination (RFE) for feature selection. By leveraging the ensemble learning technique of stacking, we enhance the model's predictive performance by combining the strengths of multiple classifiers.

**Results:**

The evaluation metrics results demonstrate the superior accuracy and obtained the higher performance in terms of accuracy, 99.25%. The effectiveness of our proposed system compared to baseline models.

**Discussion:**

Furthermore, the utilization of this system within IoT-enabled healthcare systems shows promising potential for improving heart disease diagnosis and ultimately enhancing patient outcomes.

## 1 Introduction

Heart disease (HD) is a serious public health problem that has affected millions of individuals worldwide according to the World Health Organization (WHO) (1, 2). Shortness of breath, muscle weakness, and swelling feet are prominent signs of HD (3). The diagnosis of HD is significantly important for patient treatment and recovery in the Medical Internet of Things system (MIoT) (4). Experts and medical specialists in MIoT systems have presented many non-invasive approaches for classifying and diagnosing cardiac disease (5). Machine learning (ML) and deep learning (DL) models are widely utilized in the design of computer-aided diagnosis systems (CAD) for the detection of heart disease (6).

Different heart disease diagnosis methods have been presented utilizing ML learning approaches in the literature. Detrano et al. (7) created an HD classification system utilizing ML algorithms. The Cleveland heart disease (CHD) dataset was used with global evolutionary and feature selection methods. Their proposed method recorded an accuracy of 77%. Humar et al. (8) proposed an HD detection method using a Neural Network (NN) and Fuzzy logic (FL). The classification accuracy of the said model was 87.4%. Palaniappan et al. (9) proposed a diagnosis method for HD diagnosis. The system was developed using ML models including Navies Bays (NB), Decision Trees (DT), and Artificial Neural Network (ANN). NB attained 86.12% accuracy, ANN achieved 88.12% accuracy, and 80.4% accuracy gained by the DT algorithm. Olaniyi et al. (10) proposed a three-phase model using the ANN for HD detection in angina that obtained an accuracy of 88.89%.

For the diagnosis of HD, Samuel et al. (11) designed an integrated model based on an ANN and Fuzzy AHP. In terms of accuracy, 91.10% was gained by the technique. Liu et al. (12) suggested a high-definition model based on Relief and rough set techniques. Their proposed method attained an accuracy of 92.32%. Mohan et al. (13) proposed an HD detection method using mixed ML algorithms. He also proposed a new strategy for selecting key features from data for effective machine learning classifier training and testing. They achieved 88.07% accuracy. Haq et al. (14) Proposed a machine learning-based diagnosis technique for identifying HD. ML models were used to detect HD. To choose the features, feature selection algorithms were utilized. For feature selection, they designed the Fast-Conditional-Mutual-Information (FCMIM) feature selection method. The proposed model (FCMIM-SVM) obtained a high accuracy of 92.37%. Tiwari et al. (15) proposed an ensemble approach for predicting cardiovascular illness. The framework (SE) employs a stacked ensemble classifier with machine learning algorithms such as ExtraTrees Classifier, Random Forest, and XGBoost. They have used different evaluation metrics for the proposed model (SE) evaluation. The proposed method obtained 92.34% accuracy.

The presented literature on the existing HD diagnosis models is shown in Table 1 in order to reach the problem gap in existing models in a systematic way. All of the prior treatments used a variety of methodologies to detect HD in its initial stages. However, all existing algorithms have low accuracy and are computationally complex to diagnose HD. The prediction accuracy of the HD detection approach, as shown in Table 1, requires significant enhancement for efficient and accurate detection of HD. Thus, the key concerns with the preceding methodologies are low accuracy and long computation times, which may be attributed to the usage of irrelevant features in the dataset. To solve these difficulties, new ways of identifying HD in IoT healthcare systems are necessary. Improving forecast accuracy is a major challenge and study area. Thus, the primary goal of this research is to develop an accurate and efficient HD diagnosis system.

**Table 1:** Proposed models summary.

**Model**	**FS**	**Data set**	**Acc (%)**	**Ref**
ML algorithms	–	CHD	77	(7)
MLP + SVM	–	CHD	80.41	(16)
Hybrid MLmodel (HRFLM)	–	CHD	88.07	(13)
ANN + Fuzzy Logic (ANN-FL)	–	PID and CHD	87.4	(8)
ANN ensemble-based diagnosis system	–	CHD	89.01	(17)
IHDPS	–	–	88.12	(9)
3-phase technique using ANN	–	SCH.	88.89	(10)
XGBoost		CDHD	87.28	(18)
ANN-FUZZY-AHP	–	CHD	91.1	(11)
CART		HDD	87	(19)
RRS-HD	RFRS feature selection	SCH	92.32	(12)
HISFP	Relief, mRMR, LASSO	CHD	89	(2)
SVM		Cleveland Clinic dataset	96	(20)
FCMIM-SVM	Relief, mRMR, LASSO, and LLBFS	CHD	92.37	(14)
SE		Hungarian, Cleveland, Long Beach VA, Switzerland, and Statlog	92.34	(15)

In this research study, we have proposed an ML-based computer-aided diagnosis (CAD) approach for detecting HD early in the Medical Internet of Things (IoT) system. The objective is to develop a robust and efficient system that can assist healthcare professionals in accurately identifying HD in patients. In the designing of the CAD system, we applied data pre-processing techniques such as standard scalar and the removal of null values from the data set. To select related features from the data set, we incorporated the Recursive Feature Elimination (RFE) algorithm. This helps to balance the data for proper training of the algorithm and enhance the algorithm's predictive capability. The machine learning classifiers Random Forest (RF) and Ada Boost (AB) were used for the classification of affected and healthy control subjects. These models were trained and evaluated using the entire data set and selected feature data set. To further improve the predictive results of these models, we incorporated a stacking approach to select the best meta-classifier between the Random Forest and Ada Boost. We defined a parameter grid for grid search for both algorithms. Furthermore, a hold-out validation mechanism was utilized, and data were split for training and testing in portions of 80 and 20%, respectively. The Cleveland Heart Database was used to validate the proposed model. Different performance assessment metrics were computed for model evaluation. The experimental results unequivocally demonstrated that our proposed model outperformed the baseline models in terms of predictive accuracy. Furthermore, its ease of use and compatibility with IoT healthcare systems make it an appealing and practical choice for heart disease prediction.

The innovative points of this research study are listed below:

- A CAD approach based on ML is designed to detect cardiac disease in its early stages in the MIoT systems.
- To normalize the dataset, we incorporated data preprocessing such as stander scalar and RFE algorithm for irrelevant feature elimination. The Random Forest and Ada Boost were trained and tested on entire selected feature datasets to classify heart disease and healthy control subjects.
- To further improve classification performance, the ensemble learning technique stacking was used to select the best meta-classifier between Random Forest and Ada Boost. The meta-classifier RF was used for the final classification.
- The proposed model performance was compared with baseline models, and our approach outperformed them. Hence, it is recommended for use in diagnosing heart disease in MIoT systems.

The structure of the remaining sections includes data collection and model methodology (Section 2), experiments (Section 3), discussion (Section 4), and conclusion (Section 5).

## 2 Research design

### 2.1 Data sets

The Cleveland heart disease dataset (CHD) (https://www.kaggle.com/datasets/aavigan/cleveland-clinic-heart-disease-dataset) is being examined for testing purposes in this study. Furthermore, for cross-validation of the models, we incorporated the data set Heart Statlog Cleveland Hungary (SCH) (https://ieee-dataport.org/open-access/heart-disease-dataset-comprehensive).

### 2.2 Methodology

The proposed methodology is described in the following subsections:*1) Recursive Feature Elimination (RFE) algorithm for feature selection*: feature selection is the process of selecting a subset of relevant features from a larger set of available features in a dataset. It is a critical step in machine learning and data analysis, as it helps improve model performance, reduce overfitting, and enhance interpretability. Feature selection also reduces the computation time of machine learning Algorithm 1. REF is a feature selection technique commonly used in machine learning to identify the most relevant features in a dataset. It aims to find the subset of features that are most relevant to a given machine learning task. It starts by taking a feature matrix *X* of shape (*n* samples, *n* features) and a target variable *y* of shape (*n* samples) as input. Additionally, a machine learning model is chosen to perform the feature selection process.

**Algorithm 1:**
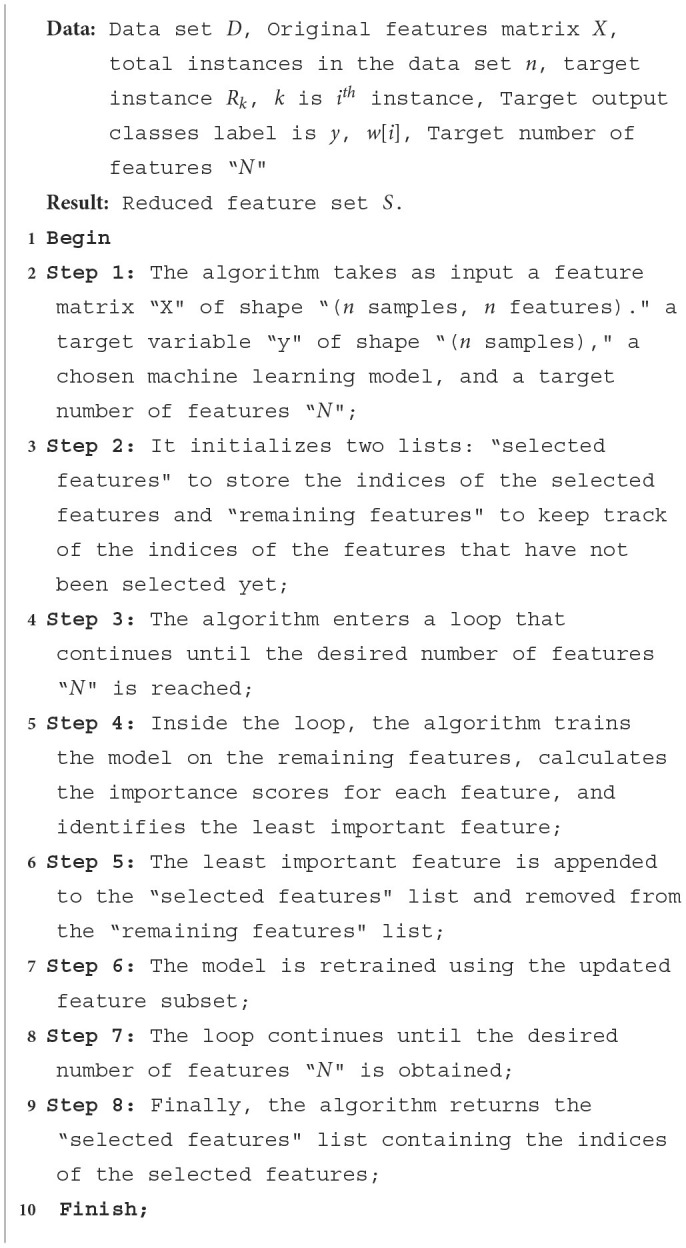
Recursive Feature Elimination (RFE) algorithm.

The RFE algorithm begins by initializing an empty list called “selected features” to store the indices of the selected features. It also creates another list of remaining features, which initially contains all the indices of the features in the “original feature” matrix.

The algorithm enters a white loop that continues until the number of selected features in selected features reaches the desired target number of features *N*. Inside the loop, the model is trained using the trained model and gets importance scores procedure.

This procedure fits the model on the subset of features given by X [: remaining features] and *y*. It then calculates the importance scores for each feature using a specific method provided by the chosen model. The importance scores represent the relevance or contribution of each feature to the model's performance.

Next, the algorithm utilizes the least important feature procedure to identify the index of the least important feature based on the importance scores. This feature is then appended to the selected feature list and removed from the remaining feature list. The algorithm proceeds by selecting the subset of features from the original feature matrix X using the indices in the selected feature list, resulting in a new matrix called X selected. The model is then retrained using this reduced feature set by applying the train model procedure, which fits the model on selected X and *y*. The loop continues until the number of selected features reaches the target number *N*. At this point, the algorithm terminates, and the selected features list contains the indices of the optimal feature subset, according to the RFE algorithm. The RFE algorithm offers several advantages, including improved model interpretability, enhanced generalization capabilities, and reduced overfitting. By iteratively eliminating the least important features and retraining the model, RFE enables the identification of the most informative features for the given task, leading to more accurate and efficient models.

Pseudo-code for the Recursive Feature Elimination (RFE) algorithm is shown in Algorithm 1.

### 2.3 Proposed classification algorithms

#### 2.3.1 Random Forest ensemble learning algorithm

Random Forest (RF) (21) is an ensemble learning algorithm that combines multiple decision trees to make predictions. It is widely used for classification and regression tasks in machine learning. The algorithm creates subsets of the original dataset through bootstrapping and constructs decision trees by recursively partitioning the data based on feature splits. The final prediction is determined by aggregating the predictions of all the trees in the ensemble. Random Forest is known for its robustness against overfitting, ability to handle large datasets, and feature importance estimation. However, it can be computationally expensive and less interpretable compared with single decision trees. The hyperparameters with essential values of random forest are shown in Table 2.

**Table 2:** Random Forest hyperparameters with essential values.

**Parameters name**	**Description**	**Values**
*N* estimators	Number of decision trees in the forest	1,000
Max depth	Maximum depth of each decision tree	20
Min samples split	Minimum number of samples required to split an internal node	10
Min samples leaf	Minimum number of samples required to be at a leaf node	5
Max features	Maximum features to use for splitting at each node	Randomness FS
Bootstrap	A boolean indicating whether to use bootstrap samples for training	True or False
Criterion	Function to measure the quality of a split (e.g., Gini impurity, entropy)	Entropy
Class weight	Weights associated with each class in classification tasks to handle class imbalance	Balance
Random state	Random seed for reproducibility	None

#### 2.3.2 Ada Boost ensemble learning algorithm

AdaBoost (AB) (22) is an ensemble learning algorithm that puts together weak learners to form a strong classifier. It iteratively trains weak learners on weighted data, focusing on misclassified samples. The resulting prediction is a weighted combination of weak learners' predictions. AdaBoost handles complex decision boundaries and achieves high accuracy but can be sensitive to noise and outliers. The hyperparameters with essential values of Ada Boost algorithm are shown in Table 3.

**Table 3:** Ada Boost algorithm hyperparameters with essential values.

**Parameters name**	**Description**	**Values**
*N* estimators	Parameters determine the number of weak learners to be included in the ensemble	200
Learning rate	Controls the contribution of each weak learner to the final prediction	0.001
Base estimator	Parameter specifies the weak learner used in the ensemble	–
Algorithm	Determines the algorithm used to update sample weights during training	“SAMME.R”

### 2.4 Stacking model based on Random Forest and Ada Boost algorithms

The stacking approach is an ensemble technique for training several base classifiers on the same dataset. Instead of making individual predictions, the predictions of these base classifiers are combined using a meta-classifier, which is typically a model such as logistic regression, random forest, or a neural network. The meta-classifier learns to make predictions based on the outputs of the base classifiers. By combining different types of classifiers, each with its strengths and weaknesses, the stacking approach aims to leverage the diverse perspectives and expertise of the individual classifiers to improve overall classification performance. This can lead to higher accuracy and better generalization compared with using a single classifier.

In this study, we trained two base classifiers (Random Forest and Ada Boost) using the entire training set. By using these two techniques, we aimed to introduce more diversity and variation into the ensemble. The predictions of each base model, Random Forest, and Ada Boost are then combined and used to train the meta-classifier, which in this case is also a Random Forest model.

### 2.5 Model cross validation

The model was trained and validated using the held-out cross-validation procedure (2). When the data set is large, the holdout CV is an appropriate validation approach. In this study, heart disease datasets such as CHD, CHDP, and SCH data sets were used and separated into 80% for training and 20% for model testing.

### 2.6 Performance evaluation criteria

The performance evaluation metrics (6) were used in this study to evaluate the proposed model performance. These evaluation metrics were expressed in equations mathematically Equations 1–6, respectively. TP denotes True Positive, TN denotes True Negative, FP denotes False Positive, and FN is False Negative.


(1) $$\begin{array}{l} \text{Accuracy (Acc)}=\frac{TP+TN}{TP+TN+FP+FN}\times 100 \end{array}$$

(2) $$\begin{array}{l} \text{Sensitivity (Sn)}=\frac{TP}{TP+FN}\times 100 \end{array}$$

(3) $$\begin{array}{l} \text{Specificity (Sp)}=\frac{TN}{TN+FP}\times 100 \end{array}$$

(4) $$\begin{array}{l} \text{Precision (Pr)}=\frac{TP}{TP+FP}\times 100 \end{array}$$

(5) $$\begin{array}{l} \text{F1-Score (F1-S)}=2\times \frac{Pr\times \text{Recall}}{Pr+\text{Recall}}\times 100 \end{array}$$

#### Matthews correlation coefficient (MCC):


(6) $$\begin{array}{l} MCC=\frac{T_{1}}{\sqrt{T_{2}\times T_{3}\times T_{4}\times T_{5}}}\times 100 \end{array}$$

where *T*_1_ = *TP* × *TN* − *FP* × *FN*, *T*_2_ = *TP* + *FP*, *T*_3_ = *TP* + *FN*, *T*_4_ = *TN* + *FP*, and *T*_5_ = *TN* + *FN*.

#### Area under the ROC curve AUC:

The AUC represents the model's ROC, and a high AUC number indicates a high-performance model. These equations represent various performance metrics commonly used in binary classification tasks.

### 2.7 Proposed model (stacking HD)

**Figure 1:**
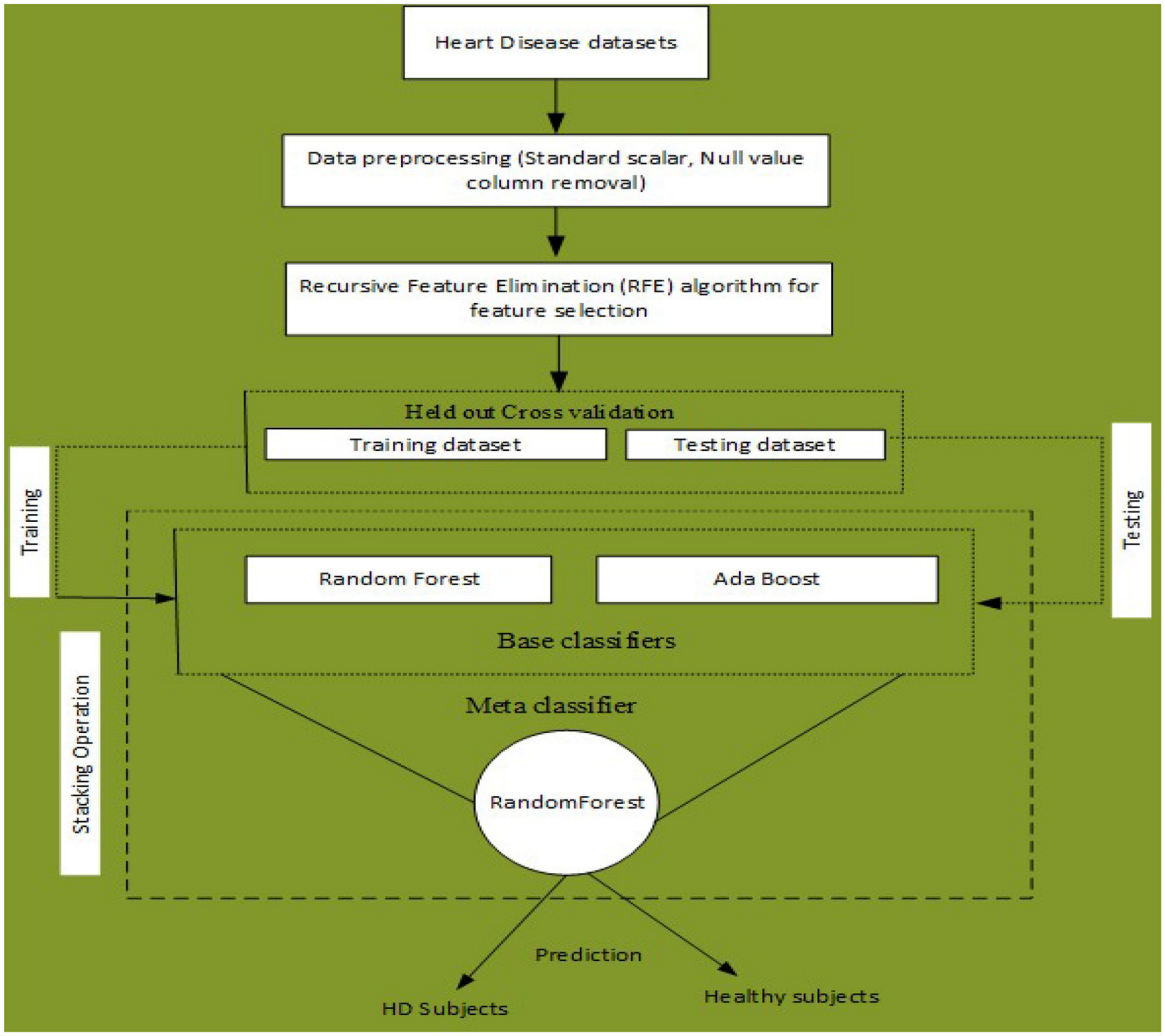
Proposed stacking-based (Stacking HD) model for Heart disease diagnosis in IoT healthcare systems.

An ML-based computer-aided diagnosis (CAD) model for detecting HD early stages in the Medical Internet of Things (IoT) system. In the designing of the CAD system, we applied data pre-processing techniques such as standard scalar and the removal of null values from the data set. To select related features from the data set, we incorporated the Recursive Feature Elimination (RFE) algorithm. This helps to balance the data for proper training of the algorithm and enhance the algorithm's predictive capability. The machine learning classifiers Random Forest (RF) and Ada Boost (AB) were used for the classification of affected and healthy control subjects. These models were trained and evaluated using the entire data set and selected feature data set. To further improve the predictive results of these models, we incorporated a stacking approach to select the best meta-classifier between the Random Forest and Ada Boost. We defined a parameter grid for grid search for both algorithms. Furthermore, a hold-out validation mechanism was utilized and data were split for training and testing in portions of 80 and 20%, respectively. The Cleveland Heart Database was used to validate the proposed model. Different performance assessment metrics were computed for model evaluation. The experimental results unequivocally demonstrated that our proposed model outperformed the baseline models in terms of predictive accuracy. The model flowchart is shown in Figure 1, and the model's method in Algorithm 2 is as follows.

**Algorithm 2:**
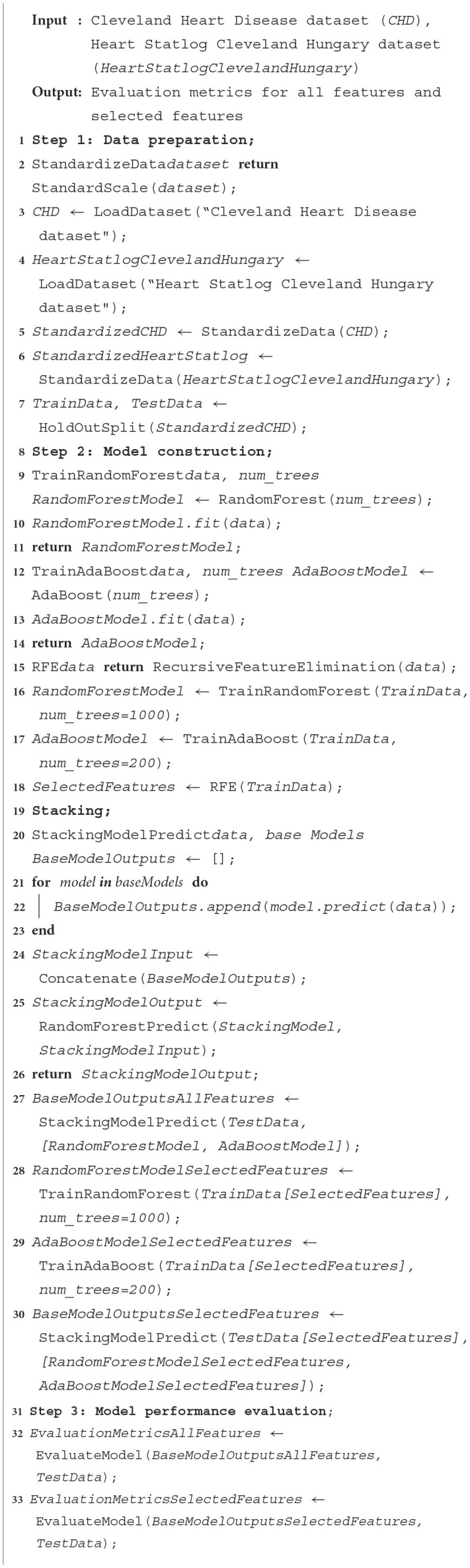
Stacking HD heart disease diagnosis.

## 3 Experiments

### 3.1 Experiments setup

For the implementation of the proposed model, we performed various experiments. First, we incorporated data preprocessing and feature selection techniques to balance the data set and remove the irrelevant features from the data set. The ML classifiers Random Forest and Ada Boost were trained on 80% the original feature data set and the selected feature data set and evaluated with 20% data. Furthermore, as shown in Tables 2, 3, additional hyperparameters were adjusted in each model accordingly. The Cleveland Heart Disease and Heart Statlog Cleveland Hungary datasets were used for validation of the models. To further improve the predictive performance, a stacking mechanism was used.

The proposed model performance was evaluated by computing various evaluation metrics. The experiments were carried out on a laptop and run with a Google collaborator accelerator. All experiments required Python v3.7 and other machine-learning libraries. Consistent values are obtained after repeating the experiments several times. The results of all experiments were provided in tables and graphed.

### 3.2 Results and analysis

#### 3.2.1 Results of data pre-processing

On the Cleveland Heart Disease dataset (CHD), the proposed model was tested. The original data set has 303 records and 75 columns; however, all published studies used only 14 columns. We did pre-processing on the data set, and 6 records were discarded due to empty values. Hence, the dataset has 297 records with 13 columns and 1 output column. As a result, a features matrix of 297*13 is created. We also employed a standard scalar to verify that each feature has a mean of 0 and a variance of 1; consequently, all features have the same coefficient. Furthermore, we duplicated 297 samples three times to increase the size of the data set. The number of samples in the new data set is 3*297 = 891. As a result, the new dataset, known as the Cleveland Heart Disease Proceeded (CHDP) data set, has a matrix size of 891*13. The description of the CHD is shown in Table 4.

**Table 4:** Description of cleveland heart disease (CHD) dataset (features matrix of 297 ^*^ 13).

**Feature name**	**Feature code**	**Feature description**
Age	AGE	Age in years
Sex	SEX	Male = 1 and Female = 0
Chest pain	CTP	Atypicalangina = 1, Typicalangina = 2, Asymptomatic = 3, Nonanginalpain = 4
Resting blood pressure	RBP	mm hg, hospitalized
Serum cholesterol	SCH	In mg/dl
Fasting blood sugar >120 mg/dl	FBS	fasting blood sugar >120 mg/dl(*T* = 1, *F* = 0)
Resting electrocardiographic	RES	Normal = 0, STT = 1, Hypertropy = 2
Maximum heart rate	MHR	–
Exercise included angina	EIA	Yes = 1, No = 0
Old peak = ST depression included by exercise relative to rest	OPK	–
Slope peak exercise St segment	PES	Up sloping = 1, Flat = 2, Down sloping = 3
Number of major vessels (0–3) colored by fluoroscopy	VCA	–
Thallium scan	THA	Normal = 3, Fixed defect = 6, Reversible defect = 7
Lable	LB	Heart disease = 1, healthy = 0

For cross-validation of the models, we incorporated the data set Heart Statlog Cleveland Hungary (SCH). This dataset has 1,190 samples with 11 columns. These datasets were collected and put in one place to enhance research on CAD-related machine learning and data mining methods and perhaps eventually advance clinical diagnosis and early treatment. The feature set Statlog Cleveland Hungary data set is shown in Table 5. The models were trained with Cleveland Heart Disease of feature matrix dataset 297*13 and 3*297 = 891 and tested with Heart Statlog Cleveland Hungary data set.

**Table 5:** Description of Statlog Cleveland Hungary (SCH) data set (features matrix of 1,190 ^*^ 11).

**Feature name**	**Feature code**	**Feature description**
Age	AGE	Age in years
Sex	SEX	Male = 1 and female = 0
Chest pain	CTP	Atypical-angina = 1, typical-angina = 2, Asymptomatic = 3, Non-anginal-pain = 4
Resting blood pressure	RBP	mm hg, hospitalized
Serum cholesterol	SCH	In mg/dl
Fasting blood sugar >120 mg/dl	FBS	fasting blood sugar >120 mg/dl (*T* = 1, *F* = 0)
Resting electrocardiographic	RES	Normal = 0, STT = 1, Hypertropy=2
Maximum heart rate	MHR	–
Exercise included angina	EIA	Yes = 1, No = 0
Old peak = ST depression included by exercise relative to rest	OPK	–
Slope peak exercise St segment	PES	Up sloping = 1, flat = 2, down sloping = 3
Targrt	TG	Heart disease = 1, healthy = 0

#### 3.2.2 Results of REF algorithm and feature ranking and selected feature subsets from CHD and SCH data sets

To choose the optimal collection of features from the SCH and CHD data sets, the REF FS method was utilized. Table 6 shows the feature rating and selected feature sets. According to Table 6, these feature sets have a significant influence on the classification of HD and HC control subjects. From CHD data set, the subset of selected features included SEX, CTP, EIA, PES, and VCA. While from SCH data set, the selected subset of features are SEx, CTP, FBG, EIA, and PES. We have performed experiments on full and selected feature datasets of both data sets in the coming sections in order to check the models' results on full and selected feature sets.

**Table 6:** Feature ranking and selected feature subsets from CHD and SCH data sets by REF algorithm, i.e., 297 * 5 ⊂297*13 and 1, 190*5⊂1, 190*11.

**Dataset**	**Feature name**	**Feature code**	**Feature ranking**	**Selected feature**
CHD	Age	AGE	7	
	Sex	SEX	1	SEX
	Chestpain	CTP	1	CTP
	Resting blood pressure	RBG	8	
	Serum cholesterol	SCH	9	
	Fasting blood sugar	FBG	2	
	Resting electrocardiographic	RES	5	
	Maximum heart rate	MHR	6	
	Exercise included angina	EIA	1	EIA
	OldPeak	OPK	3	
	SlopofST	PES	1	PES
	Flouroscorpy	VCA	1	VCA
	Thal	THA	4	
SCH	Age	AGE	6	
	Sex	SEX	1	SEX
	Chestpain	CTP	1	CTP
	Resting blood pressure	RBG	5	
	Serum cholesterol	SCH	7	
	Fasting blood sugar	FBG	1	FBG
	Resting electrocardiographic	RES	3	
	Maximum heart rate	MHR	4	
	Exercise included angina	EIA	1	EIA
	OldPeak	OPK	4	
	SlopofST	PES	1	PES

#### 3.2.3 Results of Random Forest and Ada Boost with full and selected feature data sets

The classification performance of Random Forest and Ada Boost was evaluated on whole and selected feature datasets of CHD, CHDP, and SCH datasets, respectively. The models were configured with basic hyperparameters, as shown in Tables 2, 3. The held-out cross-validation was incorporated, and data sets were divided into 80 and 20% ratios for training and validating of the models, respectively. The model's performance was evaluated by computing different evaluation metrics, and the results were reported and discussed in detail.

**Figure 2:**
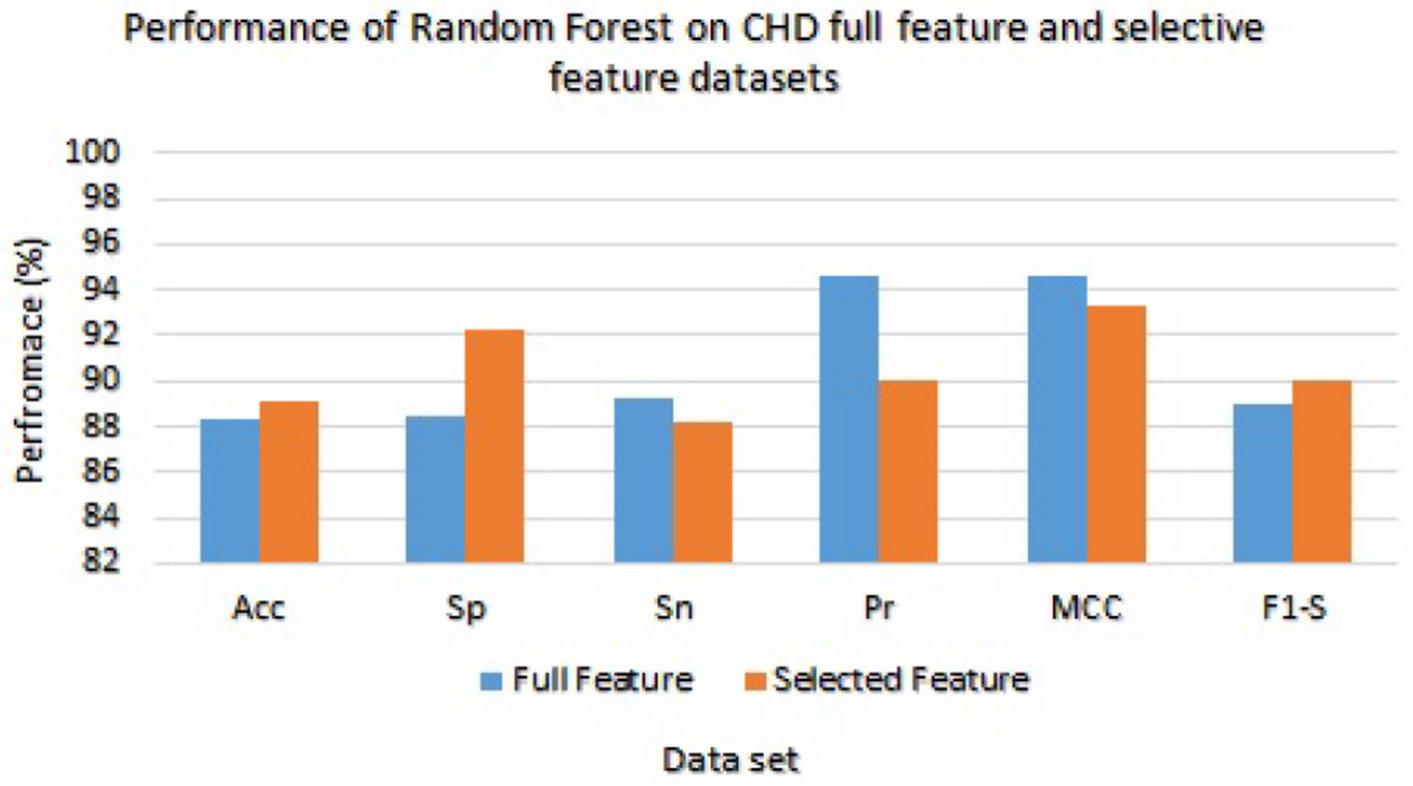
Results of Random Forest with full and selected feature sets of (CHD) data set.

Table 7 presented the results of classifiers Random forest and Ada boost trained and evaluated on full and selected feature sets on the CHD data set. On the full feature set, obtained results are 88.33% accuracy, 88.45% specificity, 89.23% sensitivity, 94.65% precision, 91.02% MCC, and 89.02% F1-score. While on selected features set the model 89.12%, 92.24%, 88.22%, 89.98%, 93.24%, and 90.00%, respectively. The model improved accuracy 89.12–88.33 = 0.79% on the selected feature set. The performance of other metrics also greatly improved. In Figure 2, Random Forest results are graphically presented.

**Table 7:** Results of Random Forest and Ada Boost with full and selected feature sets of CHD data set.

**Model**	**Data set**	**Metrics**					
		**Acc (%)**	**Sp (%)**	**Sn (%)**	**Pr (%)**	**MCC (%)**	**F1-S (%)**
Random forest	Full feature	88.33	88.45	89.23	94.65	91.02	89.02
–	Selected feature	89.12	92.24	88.22	89.98	93.24	90.00
Ada boost	Full feature	78.33	78.21	92.11	89.34	91.00	79.21
–	Selected feature	78.78	97.23	88.65	93.36	92.02	80.58

**Figure 3:**
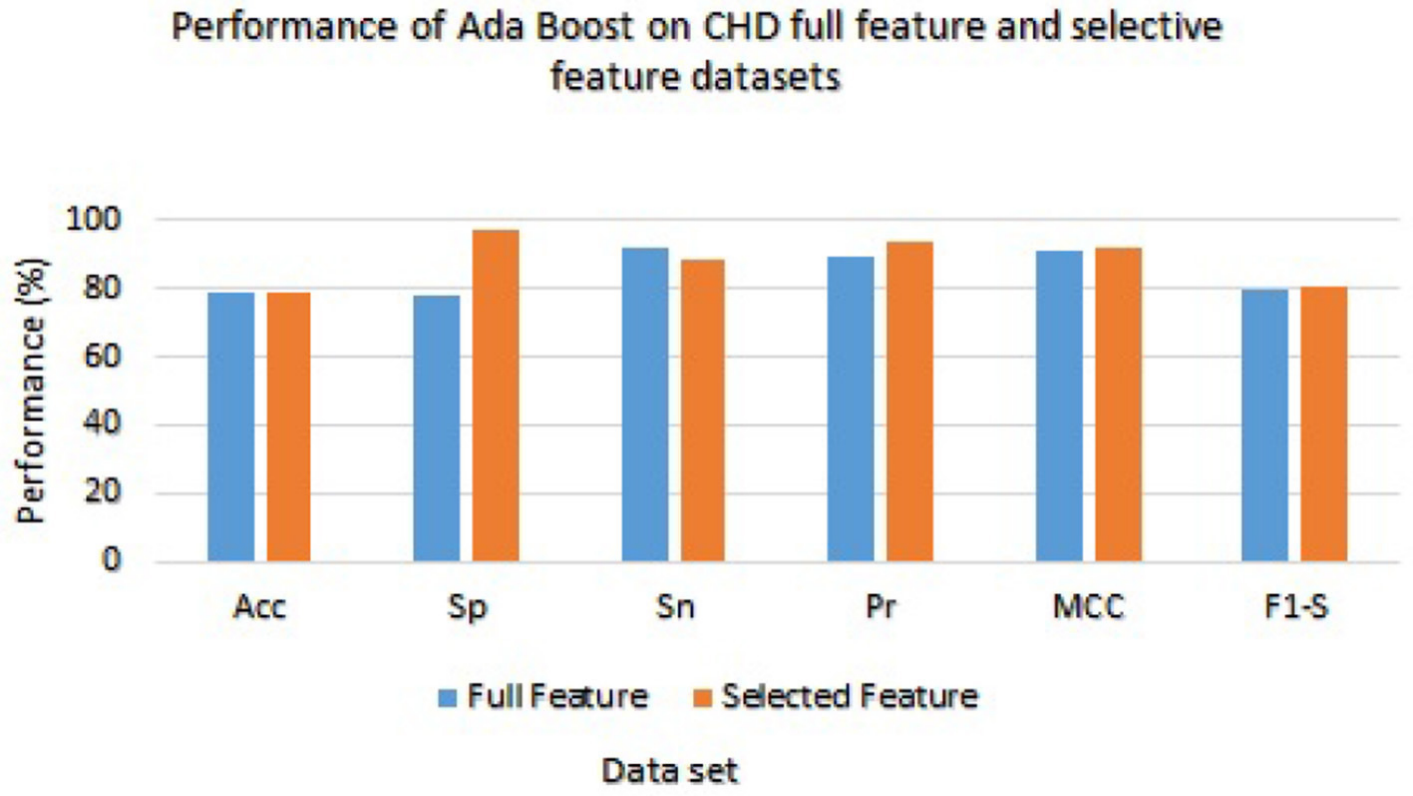
Results of Ada Boost with full and selected feature sets of (CHD) data set.

The Ada Boost results are presented in Table 7 with the full feature set and obtained 78.33%, 78.21%, 92.11%, 89.34%, 91.00%, and 79.21% of accuracy, specificity, sensitivity, precision, MCC, and F1-score, respectively. On the selected feature set, the Ada Boost achieved 78.78%, 97.23%, 88.65%, 93.36%, 92.02%, and 80.58% of accuracy, sensitivity, specificity, precision, MCC, and F1-score values, respectively. Figure 3 graphically presents the model results of Ada boost on both selected and full feature data sets of CHD data set.

**Figure 4:**
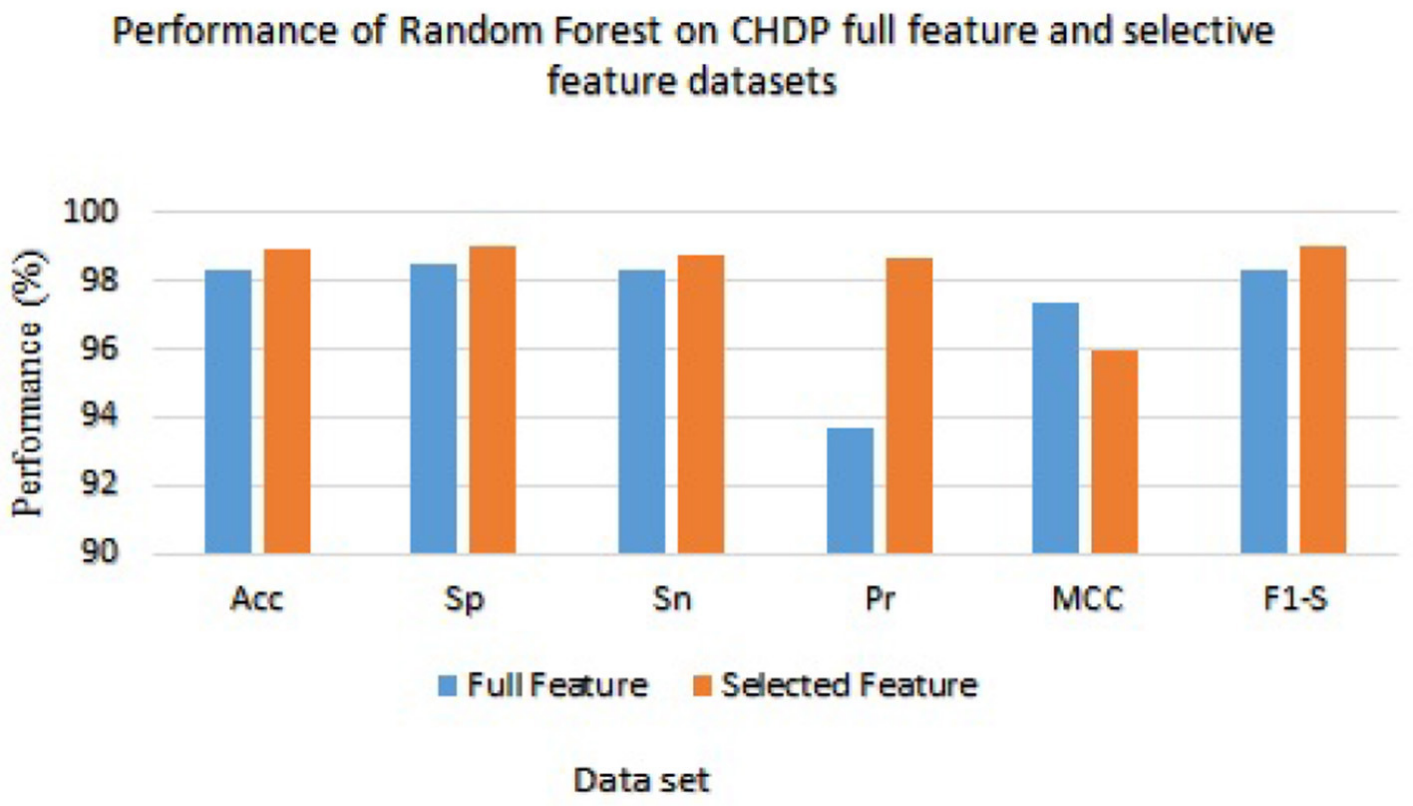
Results of Random Forest with full and selected feature sets of CHDP data set.

Table 8 presented the results of classifiers Random forest and Ada boost trained and evaluated on full and selected feature sets on the CHDP data set. The accuracy, specificity, sensitivity, precision, MCC, and F1-score values on the full feature set were 98.34%, 98.45%, 98.32%, 93.67%, 97.33%, and 98.32%, while those values on selected feature set were 98.89%, 99.00%, 98.77%, 98.67%, 96.00%, and 99.01%, respectively. The model improved accuracy 98.89–98.34 = 0.54% on the selected feature set. The performance of other metrics also greatly improved. In Figure 4, Random Forest results are graphically presented.

**Table 8:** Results of Random Forest and Ada Boost with full and selected feature sets of CHDP data set.

**Model**	**Dataset**	**Metrics**					
		**Acc (%)**	**Sp (%)**	**Sn (%)**	**Pr (%)**	**MCC (%)**	**F1-S (%)**
Random forest	Full feature	98.34	98.45	98.32	93.67	97.33	98.32
–	Selected feature	98.89	99.00	98.77	98.67	96.00	99.01
Ada boost	Full feature	93.29	93.28	93.02	94.00	93.89	94.02
–	Selected feature	93.89	93.99	94.09	95.09	96.23	94.43

**Figure 5:**
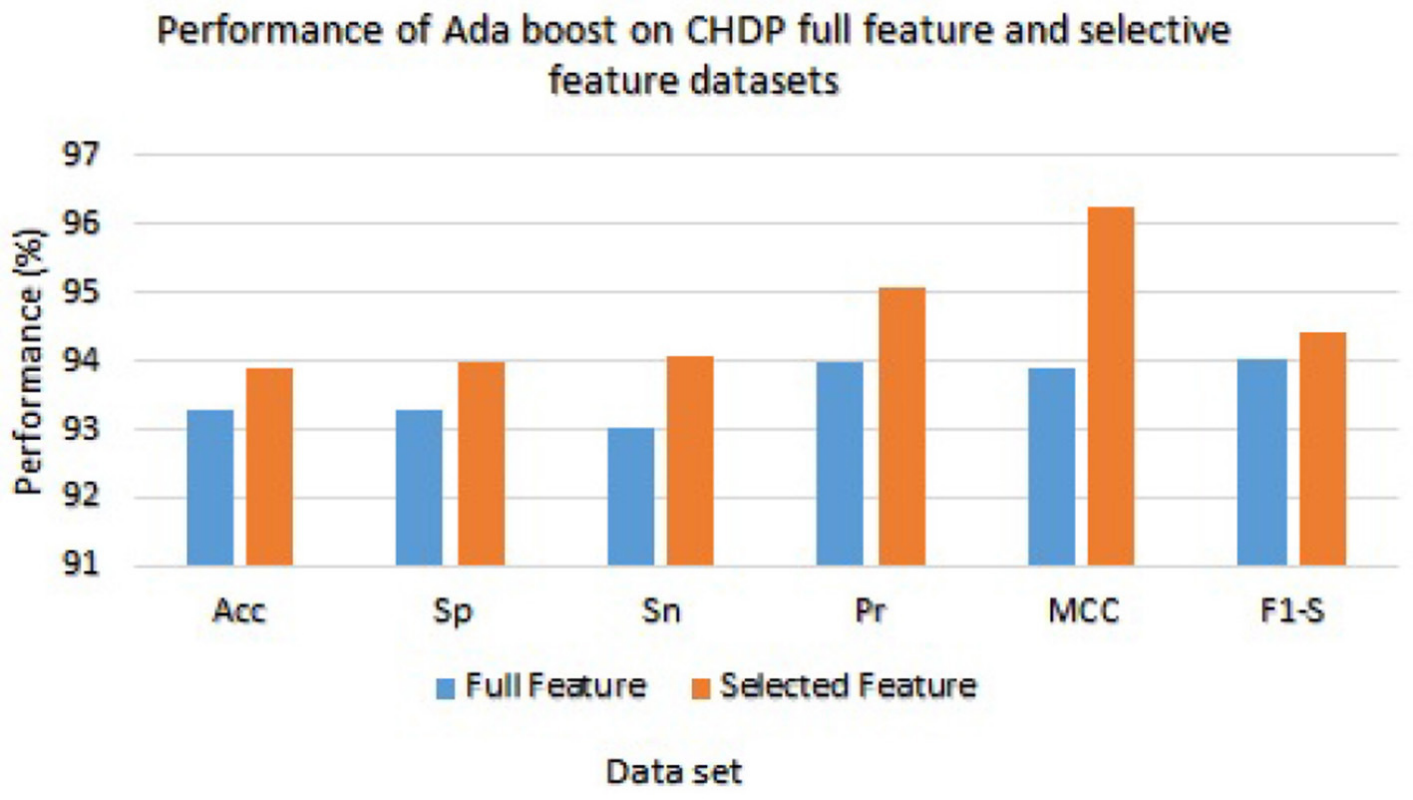
Results of Ada Boost with full and selected feature sets of CHDP data set.

On the other hand, Ada Boost results with CHDP dataset are presented in Table 8 with the full feature set and obtained 93.29% accuracy, 93.28% specificity, 93.02% sensitivity, 94.00% precision, 93.89% MCC, and 94.02% F1-score. The Ada Boost achieved 93.89% accuracy, 93.89% specificity, 94.09% sensitivity, 95.09% precision, 94.23% MCC, and 94.43% F1-measure on the specified feature set. Figure 5 graphically presented the model results of Ada boost on both selected and full feature data sets of the CHDP data set.

**Figure 6:**
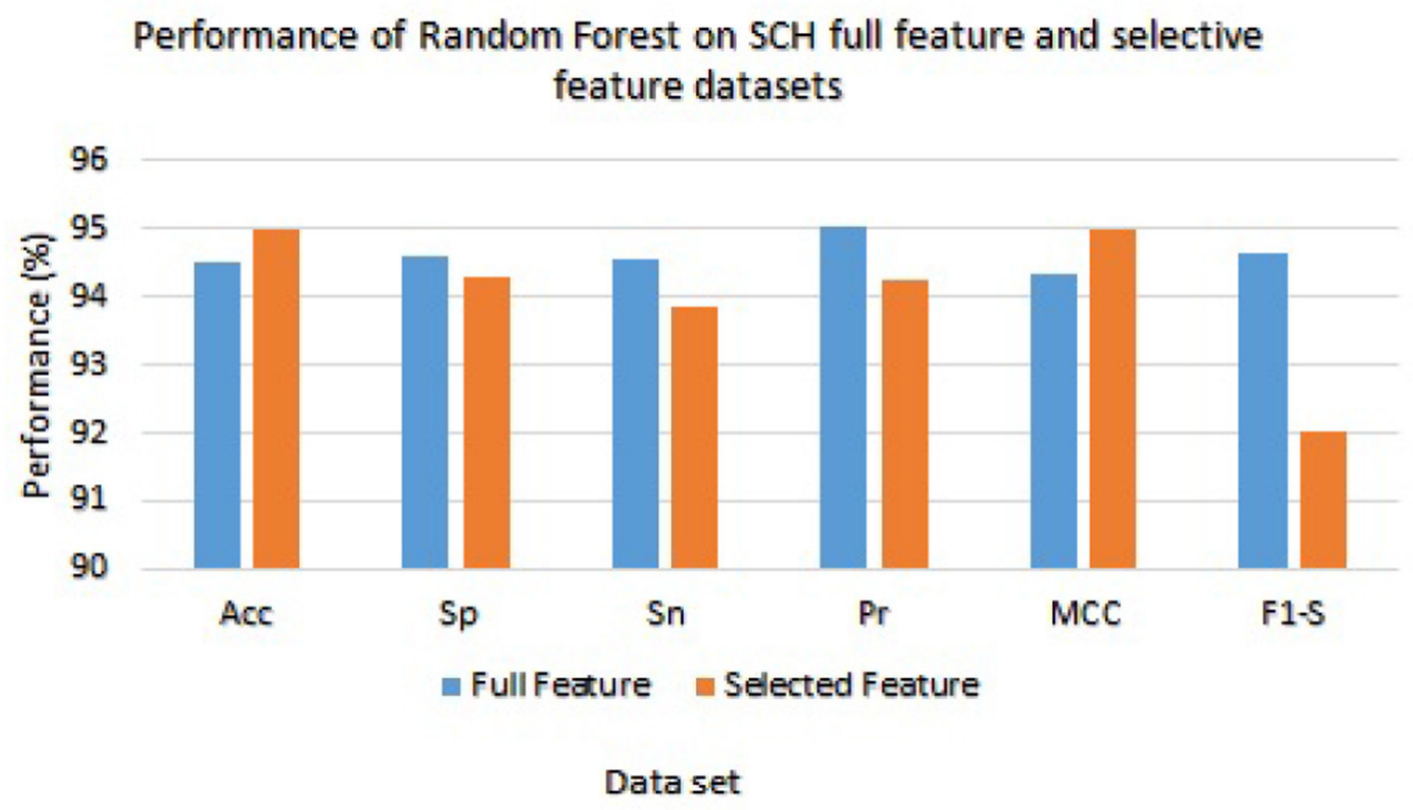
Results of Random Forest with full and selected feature sets of (SCH) data set.

We have checked the model's performance on full and selected feature data sets (SCH) in order to evaluate these models. Table 9 presented the Random Forest and Ada Boost classifier's experimental results. With the full feature set, the Random Forest gained 94.53%, 94.59%, 94.56%, 95.02%, 94.33%, and 94.53% of accuracy, specificity, sensitivity, precision, MCC, and F1-score, respectively. While accuracy, sensitivity, specificity, precision, MCC, and F1-score values on the selected feature set the Random Forest achieved 95.00%, 94.30%, 93.87%, 94.23%, 95.01%, and 92.04%, respectively. Figure 6 graphically presented the model results of Random Forest on both selected and full feature data sets of the SCH data set.

**Table 9:** Results of Random Forest and Ada Boost with full and selected feature sets of SCH data set.

**Model**	**Dataset**	**Metrics**					
		**Acc (%)**	**Sp (%)**	**Sn (%)**	**Pr (%)**	**MCC (%)**	**F1-S (%)**
Random forest	Full feature	94.53	94.59	94.53	95.02	94.33	94.53
–	Selected feature	95.00	94.30	93.87	94.23	95.01	92.04
Ada boost	Full feature	86.96	86.98	86.89	97.92	86.00	87.00
–	Selected feature	87.02	98.99	86.23	87.36	88.98	87.98

**Figure 7:**
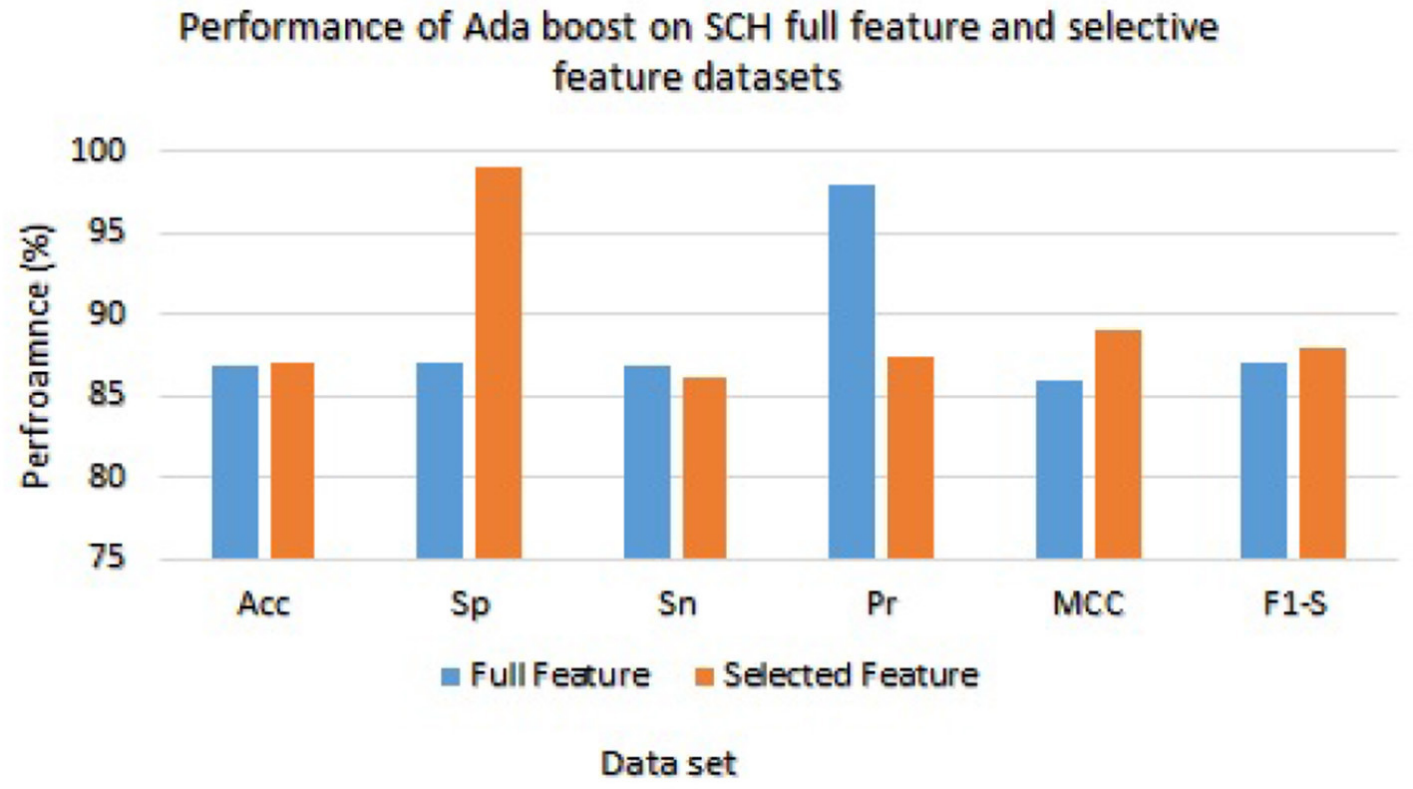
Results of Ada Boost with Full and Selected Feature sets of SCH data set.

The Ada Boost results on full and selected feature data sets (SCH) are shown in Table 9. On the full feature set, the Ada boost achieved 86.96% accuracy, 86.98% specificity, 86.89% sensitivity, 97.92% precision, 86.00% MCC, and 87.00% F1-score. The Ada Boost improved predictive performance on selected feature dataset and obtained 87.02% accuracy, 98.99% specificity, 86.23% sensitivity, 87.36% precision, 88.98% MCC, and 87.98% F1-score. Figure 7 graphically displayed the Ada Boost model results on both the selected and full feature data sets of the SCH data set.

**Figure 8:**
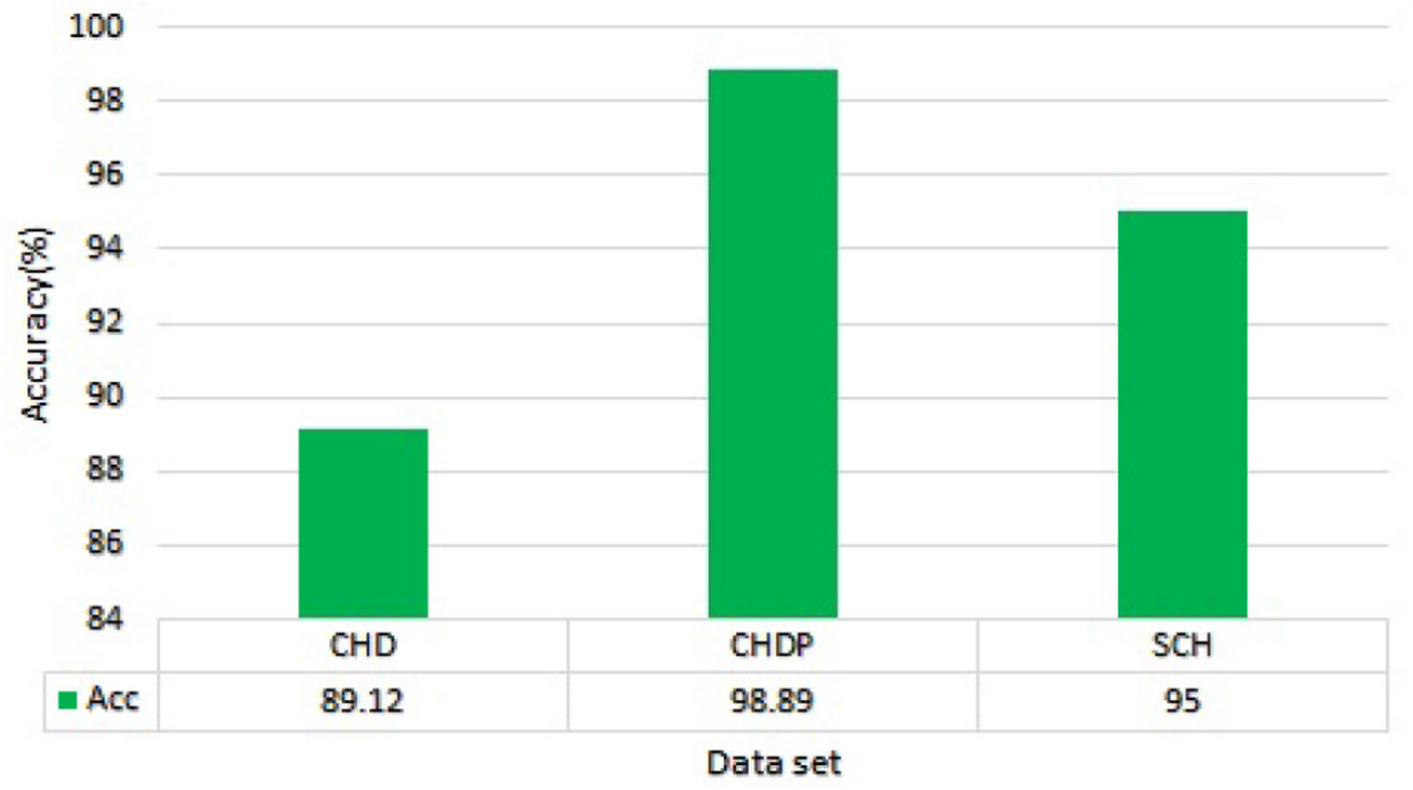
Accuracy comparison of Random Forest on three data sets.

On the basis of the experimental results of Random Forest and Ada Boost classifiers on full and selected feature sets on three datasets including, CHD, CHDP, and SCH, as shown in Tables 7–9, we concluded that the performance of Random Forest algorithm is higher as compared with Ada Boost algorithm on CHDP data set. In terms of accuracy, Random forest with CHDP data set obtained 98.89% classification accuracy. On CHD data set, the accuracy of RF algorithm was 89.12% and the accuracy of SCH data set was 95.00%. Thus, on the basis of the data set, the Random forest classifier in CHDP data set is higher than in CHD and SCH data sets. Hence, Random Forest is a suitable classifier for the diagnosis of HD in IoT healthcare systems. The RF performance in terms of accuracy on three data sets is graphically presented in Figure 8 for better understanding.

#### 3.2.4 Models performance evaluation with cross dataset

With separate cross-datasets, we examined the predictive outcomes of the Random Forest (RF) and Ada Boost (AB) classifiers. We trained the Random Forest and Ada Boost with CHD data set and tested with an independent SCH data set. The models were configured with basic hyperparameters as shown in Tables 2, 3. The model's performance was evaluated by computing different evaluation metrics and experimental results, as shown in Table 10.

**Table 10:** Classifier evaluation with cross dataset.

**Model**	**Metrics**					
	**Acc (%)**	**Sp (%)**	**Sn (%)**	**Pr (%)**	**MCC (%)**	**F1-S (%)**
Random Forest	98.97	96.87	98.73	97.24	95.28	98.70
Ada Boost	95.21	95.76	96.23	97.34	94.45	95.02

**Figure 9:**
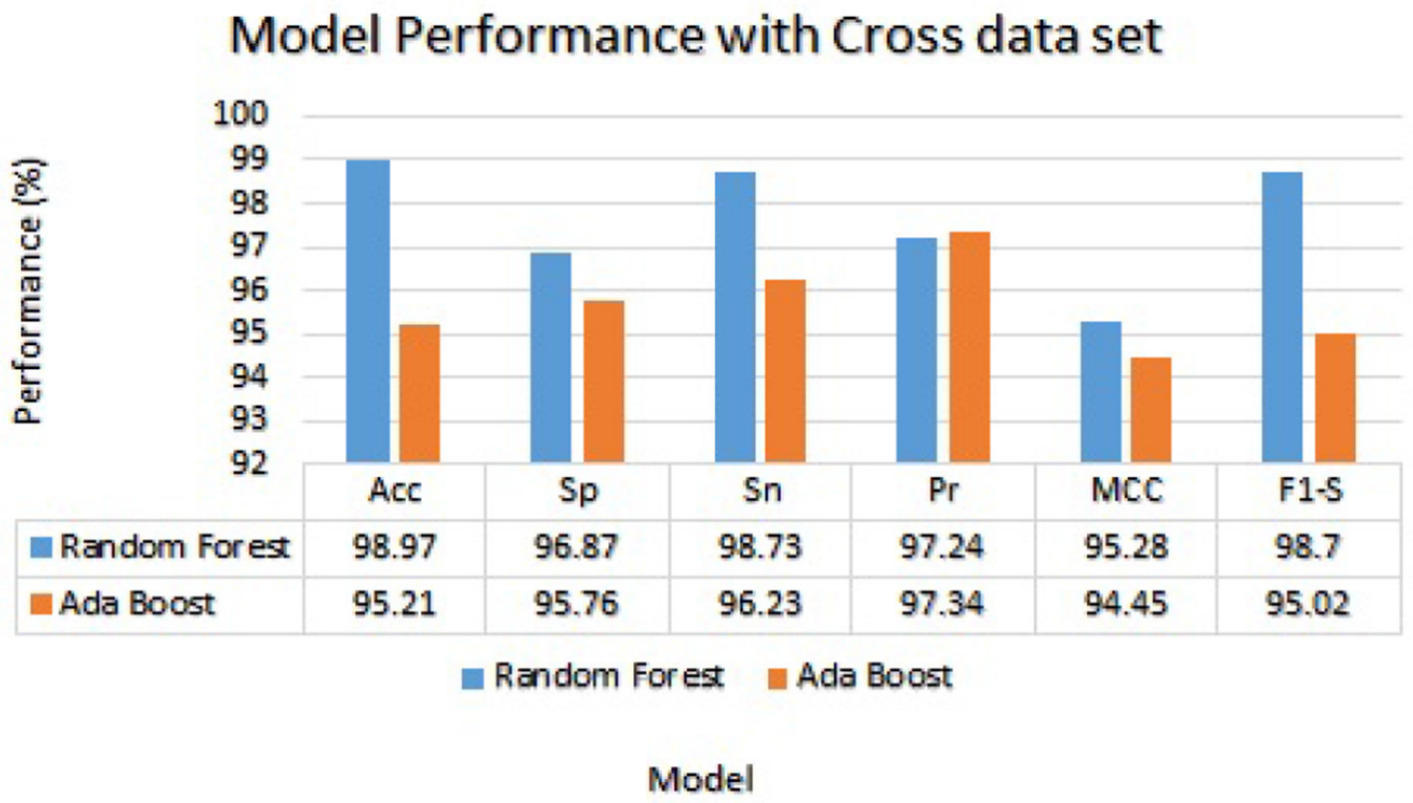
Model results trained and validated with the independent cross-data set.

Table 10 reported performance metrics results for the random forest model including accuracy, sensitivity, specificity, precision, MCC, and F1-score which were 98.97%, 96.87%, 98.73%, 97.24%, 95.28%, and 98.70%, respectively. The test accuracy of the Random forest model is higher as compared to the test accuracy of the Ada Boost model on the same data. While the Ada Boost reached an accuracy of 95.21%, a specificity of 95.76%, a sensitivity of 96.23%, a precision of 97.34%, MCC of 94.45%, and F1-score of 95.02%. The test accuracy is higher as compared to the test accuracy of the same data. The cross-data performance of Random Forest and Ada Boost is graphically shown in Figure 9.

#### 3.2.5 Results of the stacking model (stacking HD)

**Figure 10:**
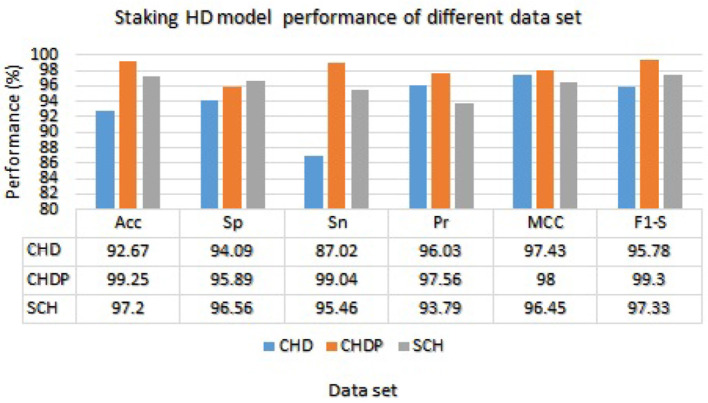
Stacking HD model performance on different data sets.

**Figure 11:**
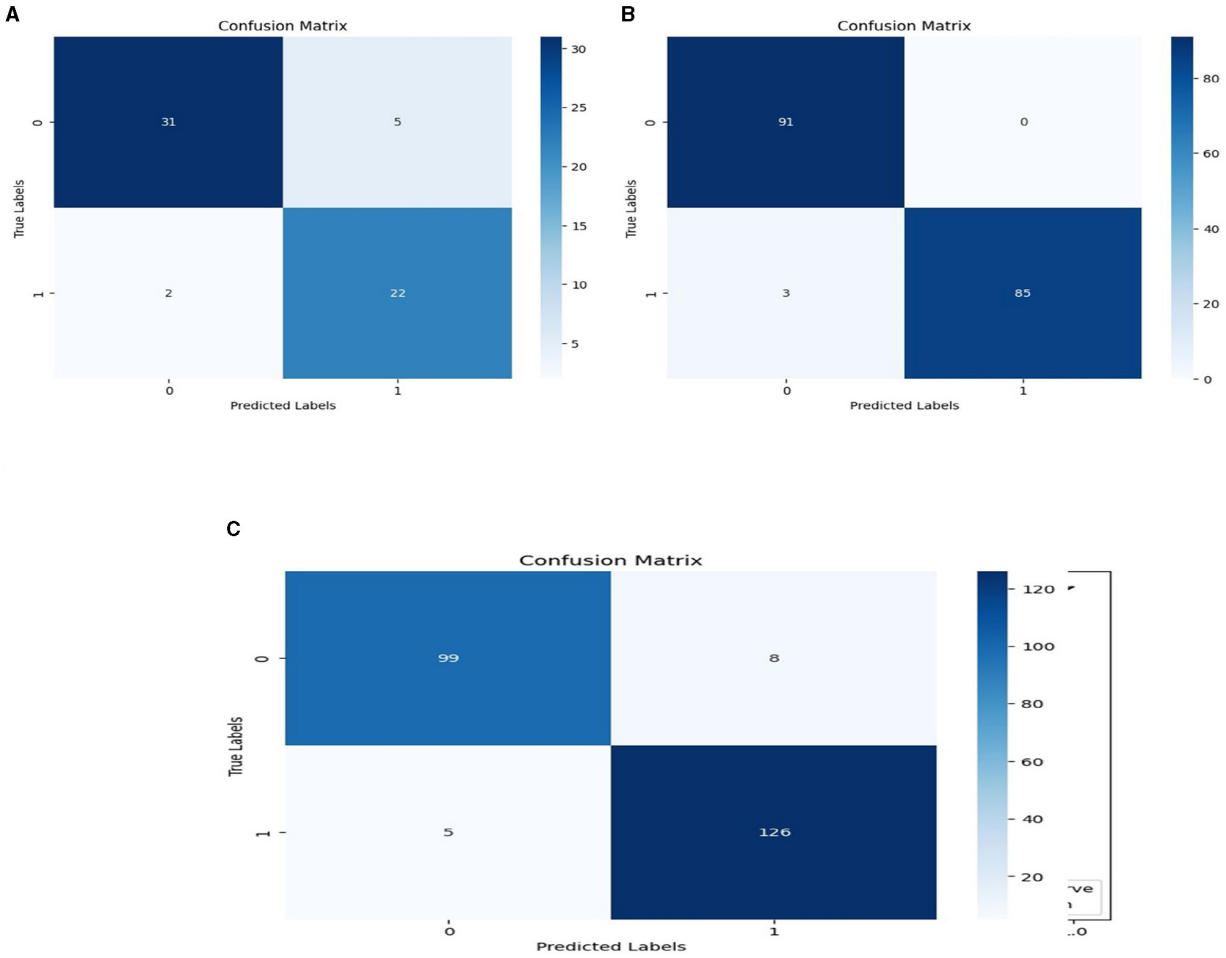
Confusion matrixes for three datasets. **(A)** Confusion matrix of staking based model on CHD data. **(B)** Confusion matrix of staking based model on CHDP data set. **(C)** Confusion matrix of staking based model on SCH data set.

**Figure 12:**
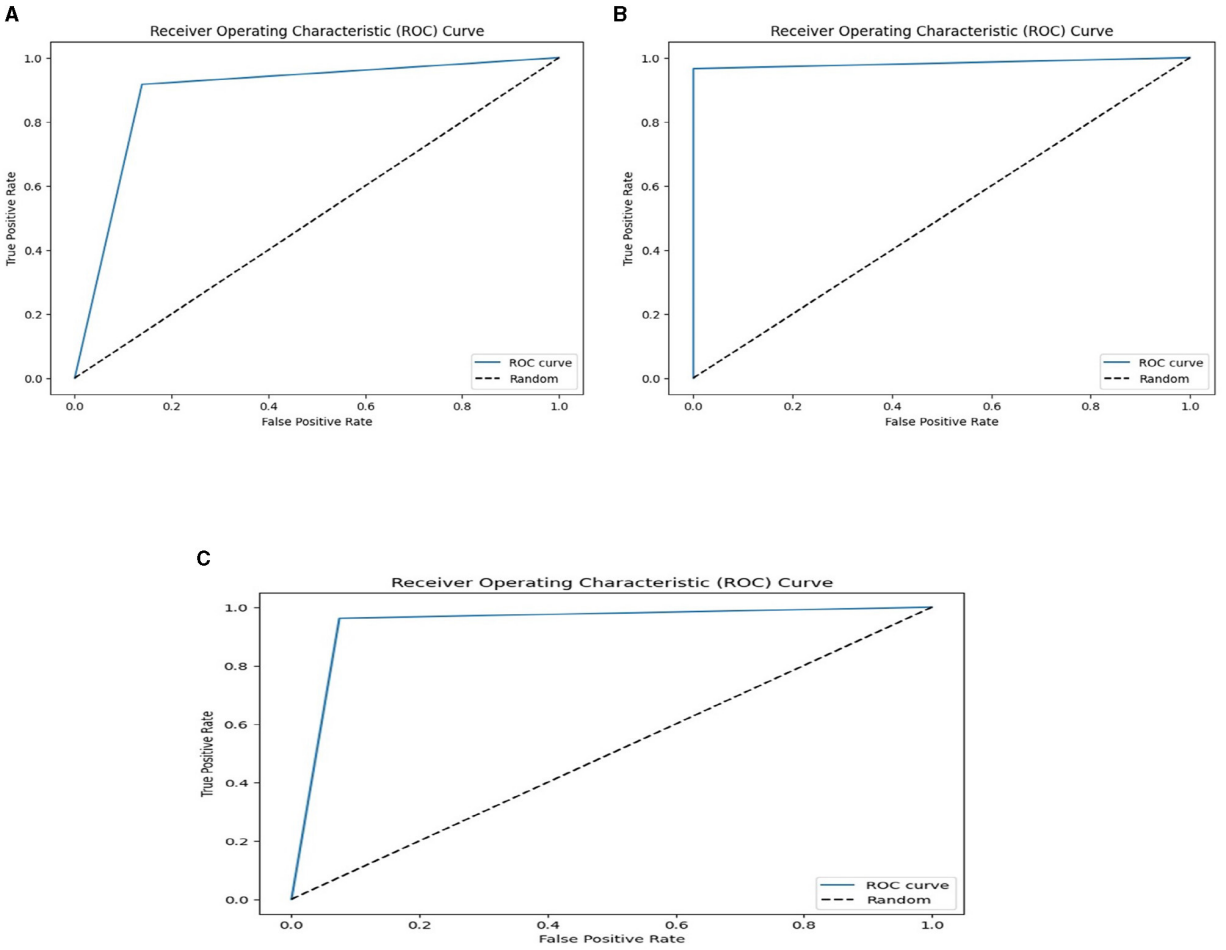
ROC curves on the stacking-based model for three data sets. **(A)** ROC curves on the stacking-based model with CHD data set. **(B)** ROC curves on the stacking-based model with CHDP data set. **(C)** ROC curves on the stacking-based model with SCH data set.

We used the performance of all models (Random Forest and Ada Boost) as new training data to increase classification performance. The Random Forest model results were highest between Random Forest and Ada Boost models when the selected feature data sets of CHD, CHDP, and SCH were used. The outcomes of the stacking-based model (stacking HD) are shown in Table 11. The stacking-based model (stacking HD) performance of different data sets is presented graphically in Figure 10 for better understanding. The table presents that the results of the stacking-based model (stacking HD) are better and obtained 92.67% accuracy, 94.09% specificity, 87.02% sensitivity, 96.03% precision, 97.43% MCC, and 95.78% F1-score on the CHD selected feature data set. The performance of the stacking approach on CHD data is better than that of individuals models Random forest as reported in Table 7 such as 89.12% accuracy, 92.24% specificity, 88.22% sensitivity, 89.98% precision, 93.24% MCC and 90.00% F1-score. The Confusion Matrix (CM) and ROC curve of the stacking-based model on CHD data set are shown graphically in Figures 11A, 12A.

**Table 11:** Stacking HD model performance with CHD, CHDP, and SCH data sets.

**Model**	**Datasets**	**Acc (%**	**Sp (%)**	**Sn**	**Pr (%)**	**MCC (%)**	**F1-S (%)**
Random Forest	CHD	92.67	94.09	87.02	96.03	97.43	95.78
Random Forest	CHDP	99.25	95.89	99.04	97.56	98.00	99.30
Random Forest	SCH	97.20	96.56	95.46	93.79	96.45	97.33

While on CHDP selected feature dataset, the stacking HD model meta classifier (Random Forest) obtained the higher performance in terms of 99.25% accuracy, 95.89% specificity, 99.04% sensitivity, 97.56% precision, 98.00% MCC, and 99.30% F1-measure. The CM and ROC curves of the stacking-based model on CHDP data set are shown graphically in Figures 11B, 12B. The stacking approach-based model on the SCH data set obtained 97.20% accuracy, 96.56% specificity, 95.46% sensitivity, 93.79% precision, 96.45% MCC, and 97.33% F1-score. The CM and ROC curves of the stacking-based model on SCH data set are shown graphically in Figures 11C, 12C. The above stacking-based model (Stacking HD) results on different data sets presented that stacking-based models perform better than individual models. The result of the stacking-based model is the high performance of CHDP data set as compared with CHD and SCH data sets. Among the three stacking model, the stacking HD on the CHDP data set obtained a higher accuracy of 99.25%. Hence, the stacking HD model is an appropriate method to diagnose HD in its early stages. Random forest is considered as the meta classifier.

#### 3.2.6 Comparison of stacking HD model with existing models

The proposed model (stacking HD) predictive accuracy is compared with baseline models, as shown in Table 12. Table 12 presented that the stacking HD model reached a higher 99.25% accuracy as compared with baseline models. The suggested method's great performance revealed that it correctly diagnoses HD and may be simply applied in IoT healthcare for the diagnosis of heart diseases.

**Table 12:** Proposed model performance comparison with baseline models.

**Model**	**Acc (%)**	**Ref**
ML algorithms	77	(7)
MLP + SVM	80.41	(16)
Random Forest model	88	(23)
GA-RBF	94.20	(24)
Hybrid MLmodel (HRFLM)	88.07	(13)
SGD	87.69	(25)
ANN + Fuzzy Logic (ANN-FL)	87.4	(8)
ANN ensemble-based diagnosis system	89.01	(17)
IHDPS	88.12	(9)
3p-ANN	88.89	(10)
RF	96.72	(26)
ANN-FUZZY-AHP	91.1	(11)
SVM	96.72	(27)
KNN	90.789	(28)
Random Forest	92.3	(29)
RRS-HD	92.32	(12)
HISFP	89	(2)
FCMIM-SVM	92.37	(14)
SE	92.34	(15)
Proposed stacking HD (CHD)	92.67	2024
Proposed stacking HD (CHDP)	99.25	2024
Proposed stacking HD (SCH)	97.20	2024

## 4 Discussion

The diagnosis of heart disease (HD) is a critical task in the early stages of IoT healthcare systems. World Health Organization (WHO) reported that a large number of people are suffered from HD each year (1). To handle the initial stages of recognition of HD, various diagnosis methods have been proposed by medical experts and researchers. Machine learning techniques based on Computer-Aided Diagnostic Systems (CAD) in an IoT healthcare system can accurately detect HD in its initial phases (30, 31). Machine learning techniques are widely used in CAD systems to diagnose critical diseases such as heart disease in IoT healthcare (32, 33). However, the existing HD diagnostic methods have the problem of lack of accuracy in the diagnosis HD correctly. The low prediction accuracy arises due to imbalanced data and irrelevant feature data for the ML model training. To address this issue, a new approach for properly and efficiently diagnosing heart disease is required for IoT healthcare systems.

The research study designed machine learning technique-based CAD systems for HD diagnosis in IoT-healthcare systems. In the designing of the CAD system, data pre-processing techniques such as standard scalar and removing null values attribute records from the data set. For related feature selection from the data set, we incorporated the Recursive Feature Elimination (RFE) algorithm to balance the data for good training of the model to enhance the model's predictive capability. The machine learning classifiers Random Forest and Ada Boost were used for the classification of affected and healthy control subjects. These models were trained and evaluated using the entire data set and selected feature data set. To further improve the predictive performance of these models, we incorporated a stacking approach to select the best meta-classifier between the Random Forest and Ada Boost. We defined a parameter grid for grid search for both algorithms.

Furthermore, the held-out validation procedure was used, and data were split into sections of 80 and 20% for training and testing. The proposed model was validated using CHD, CHDP, and SCH databases. For model performance evaluation, various performance assessment metrics results were generated. The experimental results were compared with the existing state of the arts methods.

Here, the experimental results are briefly presented. The RFE algorithm from the CHD data set of the subset of selected features included SEX, CTP, EIA, PES, and VCA. While from the SCH data set, the selected subsets of features are SEX, CTP, FBG, EIA, and PES. The performance of the Radom Forest algorithm on CHDP data was higher as compared with CHD and SCH data sets. Hence, Table 8 presented the results of the classifier Random forest trained and evaluated on full and selected feature sets on the CHDP data set. The values for the whole feature set's accuracy, sensitivity, specificity, precision, MCC, and F1-score were 98.34%, 98.45%, 98.32%, 93.67%, 97.33%, and 98.32%. While the values on selected feature set models were 98.89%, 99.00%, 98.77%, 98.67%, 96.00%, and 99.01%, respectively. The model improved accuracy 98.89–98.34 = 0.54% on the selected feature set. The performance of other metrics also greatly improved. The Random Forest accuracy is also higher than the Ada Boost classifier. Similarly, when stacking techniques were incorporated, the Random Forest performance was higher than Ada Boost, and the Random Forest model was selected as the meta-model. According to Table 7, on the CHDP chosen feature dataset, the stacking technique selected the Random Forest meta classifier and produced the higher performance in terms of accuracy, sensitivity, specificity, precision, MCC, and F1-score, each with a score of 99.25%, 95.89%, 99.04%, 97.56%, 98.00%, and 99.30%.

The confusion matrix and ROC curves of the stacking approach with data sets CHD, CHDP, and SCH are shown in Figures 11, 12. Hence, the ROC curve of the stacking model with the CHDP data set is higher, so it presents that the model accurately detected the HD as compared with CHD and SCH data sets.

Our analysis of the aforementioned results led us to the conclusion that the proposed model, stacking HD, provided better predictive outcomes and was easily implementable for HD detection in IoT-based healthcare systems.

## 5 Conclusion and future work direction

Machine learning-based Computer-Aided Diagnosis Systems are typically utilized to effectively identify heart disease. However, because current artificial diagnostic approaches are imprecise, medical practitioners are not adopting them into the heart diagnosis process efficiently. In the research study, we created an accurate technique for identifying HD using ML techniques. In the proposed approach, machine learning classifiers including Random-Forest (RF) and Ada-Boost are incorporated for the classification of heart disease and healthy control subjects. For data pre-processing and feature selection, we incorporated standard scalar and Recursive Feature Elimination (RFE) techniques to balance the data for proper training of the algorithm to enhance the model's predictive capability. We defined a parameter grid for grid search for both algorithms. To enhance algorithm accuracy, an ensemble learning technique was incorporated to select the best classification model. A held-out validation mechanism was utilized, and HD datasets were used to validate the proposed model.

The proposed model was evaluated using different evaluation metrics. According to experimental outcomes on the selected feature dataset (CHDP), the stacking technique selected meta classifier (Random Forest) and obtained the higher performance in terms of accuracy, 99.25%, and greater ROC cure. The proposed stacking HD model experimental outcomes presented that the model obtained higher results in terms of accuracy compared with existing models. Due to its excellent results, the proposed stacking HD model is recommended for HD detection in IoT healthcare systems. In the future, we will incorporate deep learning, transfer learning, and federated learning techniques to design a more advanced system for the diagnosis of heart disease in the IOT healthcare system.

## Data availability statement

The data sets utilized in this work were collected from a public repository. The Cleveland Heart Disease dataset is available at the link: https://www.kaggle.com/datasets/aavigan/cleveland-clinic-heart-disease-dataset. Heart Statlog Cleveland Hungary data set is available at the link: https://ieee-dataport.org/open-access/heart-disease-dataset-comprehensive. All methods were performed in accordance with the relevant guidelines and regulations.

## Ethics statement

Ethical review and approval was not required for the study on human participants, in accordance with the local legislation and institutional requirements.

## Author contributions

WJ: Funding acquisition, Resources, Validation, Visualization, Writing – original draft, Writing – review & editing. JL: Funding acquisition, Resources, Validation, Visualization, Writing – original draft, Writing – review & editing, Conceptualization, Data curation, Formal analysis, Investigation, Methodology, Project administration, Software, Supervision. AH: Conceptualization, Data curation, Formal analysis, Funding acquisition, Investigation, Methodology, Project administration, Resources, Software, Supervision, Validation, Visualization, Writing – original draft, Writing – review & editing. SK: Conceptualization, Data curation, Formal analysis, Funding acquisition, Investigation, Methodology, Project administration, Resources, Software, Supervision, Validation, Visualization, Writing – original draft, Writing – review & editing. RA: Writing – review & editing, Formal analysis, Visualization, Software. SA: Writing – review & editing, Methodology, Validation, Visualization, Software. MH: Data curation, Validation, Visualization, Writing – original draft, Writing – review & editing.
